# Increased Complement-Associated Inflammation in Cytomegalovirus-Positive Hypertensive Anterior Uveitis Patients Based on the Aqueous Humor Proteomics Analysis

**DOI:** 10.3390/jcm11092337

**Published:** 2022-04-22

**Authors:** Jin A Choi, Hyun-hee Ju, Jiyoung Lee, Ju-Eun Kim, Soon-Young Paik, Nikolai P. Skiba, Ponugoti Vasantha Rao

**Affiliations:** 1 Department of Ophthalmology, College of Medicine, St. Vincent’s Hospital, The Catholic University of Korea, Suwon 16247, Korea; hee8305@naver.com; 2 Department of Ophthalmology, College of Medicine, Daejon St. Mary’s Hospital, The Catholic University of Korea, Daejon 34943, Korea; ram1020@naver.com; 3 Department of Microbiology, College of Medicine, The Catholic University of Korea, Seoul 06591, Korea; brightredk@naver.com (J.-E.K.); paik@catholic.ac.kr (S.-Y.P.); 4 Department of Ophthalmology, Duke University School of Medicine, Durham, NC 27710, USA; nikolai.skiba@duke.edu (N.P.S.); p.rao@duke.edu (P.V.R.)

**Keywords:** hypertensive anterior uveitis, uveitic glaucoma, cytomegalovirus, glaucoma, inflammation, intraocular pressure

## Abstract

Herpetic anterior uveitis-associated ocular inflammation is commonly manifested with ocular hypertension and glaucoma. Relative to other viruses, cytomegalovirus (CMV) positive hypertensive anterior uveitis is associated with high recurrences of uveitis, as well as with uncontrolled intraocular pressure (IOP) and a subsequent higher requirement for future glaucoma surgery. To gain novel insights into the pathogenesis of ocular hypertension in these patients, we investigated the proteome changes of the aqueous humor (AH) derived from the CMV hypertensive anterior uveitis (CMV-HAU; *n* = 10) patients and non-glaucoma (cataract; *n* = 10) patients using liquid chromatography with tandem mass spectrometry. Among a total of 562 proteins identified, fifty and fifteen proteins were significantly elevated and decreased, respectively, in the AH of CMV-HAU patients compared to the control subjects by ≥2 fold. Gene ontology (GO) enrichment and network analyses of elevated proteins revealed that the enrichment of protein was involved in the complement activation, the humoral immune response mediated by the circulating immunoglobulins, proteolysis, and platelet degranulation. In the AH of CMV-HAU, GDF (growth/differentiation factor)-15, the inflammatory marker belonging to the TGF-β superfamily proteins, was significantly increased, while vasorin, an anti-TGF-β protein, levels were decreased. The trabecular meshwork cells infected with CMV exhibited a significantly increased expression of inflammatory markers. Collectively, these data indicate increased complement factor associated inflammation and humoral immunity in CMV-HAU associated ocular hypertension.

## 1. Introduction

Intraocular pressure (IOP) is one of the major risk factors of glaucoma, which leads to mechanical stress and compression of the nerve fiber bundle at the level of lamina cribrosa, causing the disruption of axoplasmic transport as well as microvascular circulatory disturbance [1]. Intraocular inflammation often accompanies the elevation of IOP. Among clinical entities of uveitis, anterior uveitis is the most common form and cause complicated by high IOP [2]. Hypertensive anterior uveitis is described with intraocular inflammation and elevated IOP, which can be acute, recurrent, or chronic [3]. Ocular hypertension caused by anterior uveitis often leads to glaucomatous damage in about 20–30% of the cases [4,5].

The growing body of literature indicates that cytomegalovirus (CMV) infection is a major cause of hypertensive anterior uveitis in immunocompetent patients [6,7]. CMV hypertensive anterior uveitis (CMV-HAU) is characterized by a sudden and robust elevation of IOP with the onset of inflammation, followed by the normalization of IOP with the resolution of inflammation [6,7]. Clinically, CMV-HAU is not only associated with high recurrences of uveitis and severe corneal endothelial cell damage, but also with uncontrolled IOP and a subsequent higher requirement for future glaucoma surgery compared to CMV negative hypertensive anterior uveitis [6,7,8].

Despite the clinical significance of the disease, pathophysiologic mechanisms underlying the robust but reversible elevation of IOP in CMV-HAU remain poorly understood. Virus-induced contraction of trabecular meshwork (TM) cells and increased expression of inflammatory chemokines by the virus-infected TM cells have been reported recently as plausible contributory factors for the elevated IOP in CMV-HAU patients [9,10]. However, there were no previous studies to investigate the changes in the protein composition of aqueous humor (AH) derived from the CMV-HAU patients. The comprehensive understanding of AH proteomics in CMV-HAU patients could provide novel insights into the pathogenesis of IOP elevation in these patients.

Thus, in this study, we investigated the compositional changes in the AH proteome of CMV-HAU patients in comparison with non-glaucoma (cataract) patient samples using unbiased and quantitative mass spectrometry-based proteome analyses.

## 2. Methods

### 2.1. Human Subjects

In this cross-sectional study, AH samples from immunocompetent patients with CMV-positive hypertensive anterior uveitis and adults undergoing cataract surgery were obtained from St. Vincent’s Hospital, Suwon, Catholic University of Korea. The study protocol was approved by the Review Board of St. Vincent’s Hospital, Catholic University of Korea (IRB number: VC19SESI0271). All procedures adhered to the tenets of the Declaration of Helsinki. Written informed consent was obtained from patients prior to the collection of AH. Samples were collected starting from the beginning of October 2016 to June 2019.

### 2.2. Clinical Assessment

The inclusion criteria of CMV-HAU were as follows: (1) tested positive for intraocular CMV infection based on PCR confirmation, (2) at least two acute episodes of a low-grade anterior uveitis (anterior chamber cells 1+, flare, keratic precipitates) with a hypertensive IOP, and (3) the absence of posterior synechiae or retinitis [11]. The controls had only cataracts, without any evidence of glaucoma or any other eye diseases.

All of the participants underwent a comprehensive ophthalmic examination, including a detailed review of medical and ocular histories, best-corrected visual acuity measurement, slit-lamp biomicroscopy, Goldmann applanation tonometry, and specular microscopy using a non-contact specular microscope (NSP-9900; Konan Medical, Tokyo, Japan). In patients with CMV-HAU, dilated stereoscopic examination of the optic nerve head and fundus, stereoscopic optic disc photography and red-free retinal nerve fiber layer (RNFL) photography (CF-60UD; Canon, Tokyo, Japan), achromatic automated perimetry using the 24–2 Swedish Interactive Threshold Algorithm Standard program (Humphrey Visual Field (VF) Analyzer; Carl Zeiss Meditec, Inc., Dublin, CA, USA), and optical coherence tomography scans (Cirrus OCT, Carl Zeiss Meditec) were performed to measure the peripapillary RNFL thickness.

### 2.3. Collection of Human AH

The anterior chamber tap has been routinely recommended when the aforementioned clinical criteria of CMV-HAU were fulfilled. Using a 30-gauge needle, 100 μL AH was aspirated under aseptic conditions and subjected to a polymerase chain reaction (PCR) assay for CMV. Separately, an additional 30 µL AH was collected. For controls, AH samples were collected at the initiation of cataract surgery. The supernatant (1000× *g*) obtained from the AH samples was collected and frozen at –80 °C until further analysis.

### 2.4. Mass Spectrometry

#### 2.4.1. Sample Preparation and LC-MS/MS Analysis

An equal volume (10 µL) of AH samples from cataract and CMV-HAU patients were solubilized in 2% sodium dodecyl sulfate, 100 mM Tris-HCl (pH 8.0), reduced with 10 mM dithiothreitol, alkylated with 25 mM iodoacetamide, and subjected to tryptic hydrolysis using the HILIC beads SP3 protocol (ReSyn Biosciences, Gauteng, South Africa) [12]. Each digest was dissolved in 12 μL of a 1/2/97% (by volume) trifluoroacetic acid/acetonitrile/water solution, and 3 μL was injected onto a 5 μm, 180 μm × 20 mm Symmetry C18 trap column (Waters Corp. Milford, MA, USA) run using 1% acetonitrile in water for 3 min at 5 μL/min. The analytical separation was next performed using an HSS T3 1.8 μm, 75 μm × 200 mm column (Waters) over 90 min at a flow rate of 0.3 μL/min at 55 °C. The 5–30% mobile phase B gradient was used, where phase A was 0.1% formic acid in water and phase B was 0.1% formic acid in acetonitrile. Peptides separated by liquid chromatography (LC) were introduced into the Q Exactive HF Orbitrap mass spectrometer (Thermo Fisher Scientific, Waltham, MA, USA) using positive electrospray ionization at 2000 V and a capillary temperature of 275 °C. Data collection was performed in the data-dependent acquisition (DDA) mode with 120,000 resolution (at *m*/*z* 200) for MS1 precursor measurements. The MS1 analysis utilized a scan from 375–1450 *m*/*z* with a target AGC value of 1.0e6 ions, the RF lens set at 30%, and a maximum injection time of 50 ms. Advanced peak detection and internal calibration (EIC) were enabled during the data acquisition. Peptides were selected for MS/MS using charge state filtering (2–5), monoisotopic peak detection, and a dynamic exclusion time of 25 s with a mass tolerance of 10 ppm. MS/MS was performed using higher-energy C-trap dissociation (HCD) with a collision energy of 30 ± 5% with detection in the ion trap using a rapid scanning rate, automatic gain control target value of 5.0e4 ions, maximum injection time of 150 ms, and ion injection for all the available parallelizable time enabled.

#### 2.4.2. Protein Identification and Quantification

For label-free relative protein quantification, raw mass spectral data files were imported into Progenesis QI for Proteomics 4.2 software (Nonlinear Dynamics) for duplicate runs, alignment of each preparation, and peak area calculations. Peptides were identified using Mascot version 2.6.2 (Matrix Science) for the searching human UniProt 2019 reviewed database containing 20,237 entrees. Mascot search parameters were: 10 ppm mass tolerance for precursor ions; 0.025 Da for fragment-ion mass tolerance; one missed cleavage by trypsin; fixed modification was carbamidomethylation of cysteine; variable modifications were oxidized methionine and Asn/Gln deamidation. Only proteins identified with two or more peptides (protein confidence *p* < 0.05 and false discovery rate < 1%), were included in the protein quantification analysis. To account for variations in experimental conditions and amounts of protein material in individual LC-MS/MS runs, the integrated peak area for each identified peptide was corrected using the factors calculated by the automatic Progenesis algorithm utilizing the total intensities for all peaks in each run. Values representing protein amounts were calculated based on a sum of ion intensities for all identified constituent non-conflicting peptides [13]. For protein profiling, we compared relative amounts of proteins confidently identified in the control and treated samples. Proteins with at least a two-fold change in abundance and ANOVA *p* scores less than 0.05 were selected as statistically significant.

The mass spectrometry proteomics data have been deposited to the MassIVE server with the data set identifier MassIVE MSV000089079; (https://urldefense.com/v3/__http://massive.ucsd.edu/ProteoSAFe/status.jsp?task=9b6a972f1e0540f2b4fffe651ffe86aa__;!!OToaGQ!4ZdfTzLNyGXiGozoUsUX01rJOa34b79K73eCbJJSI83P30iMOUjNc5WxfUIA5UiXAqtW$/ (Accessed: 24 March 2022)).

### 2.5. TM Cell Culture and CMV Virus Infection

Primary human TM cell cultures were derived from freshly obtained donor corneal rings (from two different human donors aged 39 and 16 years) upon informed consent. All eyes were enucleated within 6-hr postmortem, and tissues were explanted for culture within 24-hr postmortem. TM cells were isolated and grown in the tissue culture medium by following the whitepaper guidelines established to use human TM cells [14]. The culture medium consisted of low glucose Dulbecco’s modified Eagle’s medium (DMEM) supplemented with 15% fetal bovine serum (FBS; Invitrogen-Gibco, Grand Island, NY, USA), and 1% penicillin-streptomycin was used. Cells derived from the TM tissue were passaged and used in the described experiments at passages 3–6.

After TM cells became confluent, cells were mock-infected or infected with CMV at a multiplicity of infection (MOI) of 1, as previously described [15]. Human CMV strain AD169 was propagated using HFF (human foreskin fibroblast) cells, and virus stocks were titrated using a 50% tissue culture infectious dose (TCID50) assay on HFF cells, using the method of Reed and Muench [16]. After viruses were adsorbed for 2 h on confluent TM cells, the infected cells were washed once with 1× phosphate-buffered saline (PBS) and the culture medium was applied. At 2 days post-infection, analyses were performed using the mock-infected and CMV-infected TM cells.

### 2.6. qRT-PCR Analyses

Using RNeasy Mini Kit; Qiagen, Valencia, CA, USA), total RNA was extracted. A cDNA synthesis kit (PrimeScript RT reagent Kit, Takara, Japan) was used for first-strand cDNA synthesis. The relative expression levels of mRNA were determined using a Roche Diagnostics LightCycler 2.0 Real-Time PCR System (Roche GmbH, Mannheim, Germany) according to the manufacturer’s instructions. Real-time qPCR was conducted on the resultant reverse transcriptase-derived single stranded cDNA using sequence-specific forward and reverse oligonucleotide primers for the target genes (Supplementary Table S1), as previously described. Reactions for each sample were run in triplicate, cycle thresholds were normalized to β-actin expression, and comparative quantitation was performed (LightCycler software, version 4.1, Roche, Basel, Switzerland). Individual PCR samples with single-peak dissociation curves were selected for data analysis.

### 2.7. Enzyme-Linked Immunosorbent Assay (ELISA)

Human GDF15 (Human GDF15 ELISA, R&D Systems, Inc., Minneapolis, MN, USA) and a human Vasorin ELISA kit (Human Vasorin/SLIT-Like 2 ELISA Kit, MyBioSource, Inc, San Diego, CA, USA) were used to determine the levels of GDF15 in the AH samples of human subjects and vasorin in the condition medium of CMV infected TM cells, respectively. Analysis was performed in a masked manner, using the manufacturer’s protocol, which included appropriate standards and background controls, using a SpectraMax 190 Microplate Reader (Molecular Devices, LLC., San Jose, CA, USA). Ten microliters of AH was used from the control and CMV-HAU patients. While the duplicate samples were used for the control (non-glaucoma subjects) specimens, for the CMV-HAU patients, only a single aliquot (10 µL) of AH was used due to the scarcity of AH sample available.

### 2.8. Proteome Functional Analyses

To gain insights into the functional impact of altered protein profiles of AH derived from the CMV-HAU patients, we performed a gene enrichment analysis using GO (gene ontology) tools available in the public domain. For this, we initially used ShinyGO v0.60 for enrichment of GO terms (http://bioinformatics.sdstate.edu/go/ (Accessed: 24 March 2022)). A cutoff of *p*-value < 0.05 was adjusted for all GO categories. To understand and construct the protein–protein interaction networks, we also used protein–protein interactome information from the STRING (Search Tool for the Retrieval of Interacting Genes/Proteins) version 11.5 public database (https://string-db.org/ (Accessed: 24 March 2022)).

### 2.9. Statistical Analyses

Data were processed using the biostatistical package InStat (GraphPad, San Diego, CA, USA). Experiments were performed in triplicate, and representative results are shown. All data were expressed as mean ± standard error of the mean (SEM) values based upon analysis of at least three independent samples, until otherwise mentioned. Student’s t test was used for the cell-based studies to compare the changes between two groups (control and test). A *p* < 0.05 was considered to indicate statistical significance. The Mann–Whitney U-test was employed for comparing GDF-15 and vasorin protein levels in human AH samples from the CMV-HAU and control groups.

## 3. Results

### 3.1. Demographic and Clinical Characteristics of Human Subjects

A total of 20 AH samples were analyzed in this study, 10 from CMV-HAU patients and 10 from control (non-glaucoma) patients who underwent routine cataract surgery. Clinical data of both groups are presented in Table S2. As described in Table S2, the control group consisted of significantly older patients compared to the CMV-HAU patients. The CMV-HAU patients used 2.1 ± 1.0 bottles of anti-glaucoma eyedrops and had significantly higher peak preoperative IOP compared to the control patients (*p* < 0.001). The corneal endothelial cell count was significantly lower in patients with CMV-HAU compared to the controls (*p* < 0.015). In patients with CMV-HAU, the mean deviation of the visual field test by standard automated perimetry was −8.43 ± 6.51 dB, and the average RNFL (retinal nerve fiber layer) thickness was 78.2 ± 16.2 um.

### 3.2. Evidence for Complement Activation in CMV-HAU Patients

A total of 562 proteins were identified in the described AH samples by LC-MS/MS analysis. Upregulation and downregulation was defined as the ratio between the CMV-HAU and controls by two or greater than two-fold (≥2 fold) change. According to this definition, 50 individual proteins were upregulated and 15 individual proteins were down-regulated in the AH of CMV-HAU compared to the controls (non-glaucoma). Quantitative proteomic results are presented in Table 1 and Table 2, including fold change, mass, SEM, confidence score, and *p*-values.

**Figure 1:**
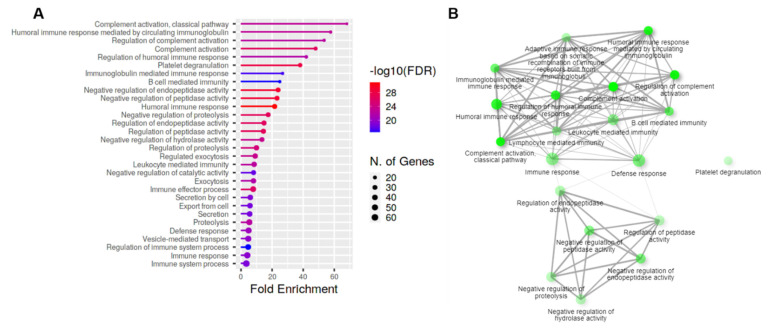
The top significant over-represented pathway after functional enrichment analyses and their interaction network analysis of the aqueous humor of the CMV hypertensive anterior uveitis for the 30 most significant terms. In the enriched pathway of the biological process (**A**), the *x*–axis indicates the number of genes represented in each pathway, the *y*–axis indicates the gene ontology of the biological process involved, and the colors represent the size of the negative log10 of the adjusted v–value (adjusted *p* < 0.05). In the interaction network analyses of the relationship between the biological process (**B**), and biological processes that are enriched by upregulated proteins are shown. Thicker edges (lines) show that there are more overlapped edges. Bigger and darker nodes represent larger protein sets and more significantly enriched proteins, respectively. The plot was generated from ShinyGO v0.75.

A total of 50 upregulated proteins were subjected to subsequent functional enrichment analyses for biologic processes. For these analyses, data were filtered using a *p*-value cutoff of 0.05 and the 30 most significant terms. GO enrichment analyses and network analyses indicated that proteins related to the complement activation, humoral immune response mediated by circulating immunoglobulin, proteolysis, and platelet degranulation were mainly enriched (Figure 1A,B). The predicted genes related to the corresponding function are shown in Table S3.

**Figure 2:**
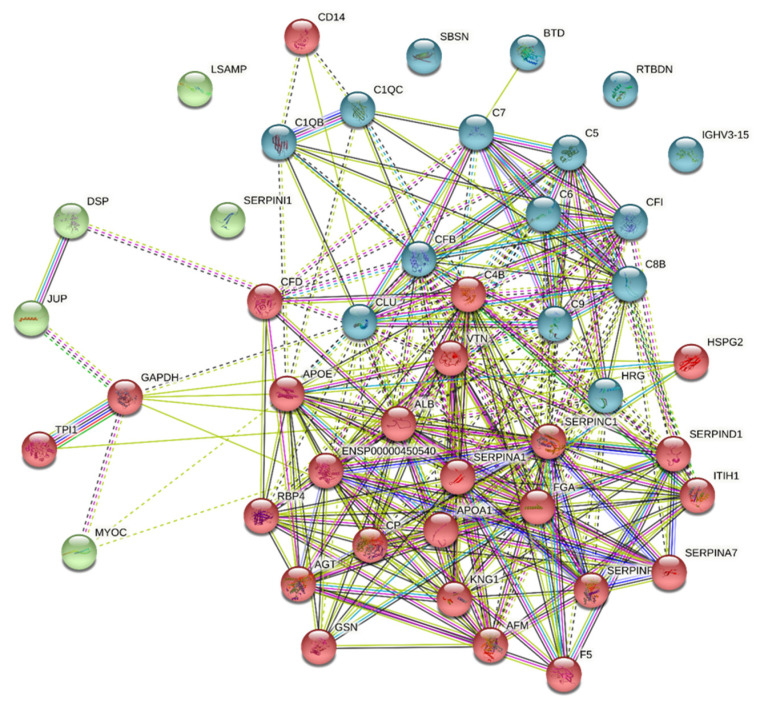
The cluster analysis of 50 differentially elevated proteins in aqueous humor from CMV hypertensive anterior uveitis. The identified clusters are colored in red, blue, and green. The solid and the dotted lines indicate a connection within the same and different clusters, respectively. Different color indicates a different type of interaction. Search Tool for the Retrieval of Interacting Genes/Proteins (http://string-db.org/ (Accessed: 24 March 2022)) was used to identify groups within them so as to understand the pathogenesis of the CMV hypertensive anterior uveitis. Cluster colored red was found to be enriched in genes regulating protein activation and phospholipid efflux. Cluster colored blue had genes that were found to be related with complement activation. Cluster colored green was very small with only 8 genes. We could not find enriched processes for this cluster; however, the genes of this cluster were involved mainly in cell adhesive protein bindings.

To identify the functional groups in the predicted genes to understand the pathogenesis of CMV-HAU associated ocular hypertension, the clustering analyses were performed on the predicted 50 genes using the K-means clustering STRING method. This analysis resulted in a network with a high clustering coefficient of 0.55 and an average node degree of ~14.2. The high clustering coefficient suggests that the network is also a community that may be involved in a similar kind of function. This analysis identified three clusters (referred to as clusters A, B, and C) with sizes of 28, 14, and 8 genes, respectively (Figure 2). Cluster A was found to be enriched in genes regulating protein activation and phospholipid efflux. Cluster B had genes that were found to be related with complement activation. Cluster C was very small with only 8 genes. We could not find enriched processes for this cluster; however, the genes of this cluster were involved mainly in cell adhesive protein bindings.

### 3.3. Increased Inflammation Markers in the AH of CMV-HAU Patients

**Figure 3:**
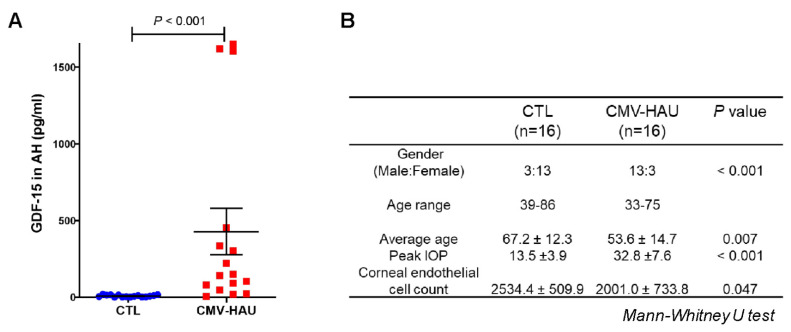
The levels of GDF–15 in the aqueous humor of the CMV hypertensive anterior uveitis patients from ELISA. (**A**) Aqueous humor (*n* = 16) samples derived from CMV hypertensive anterior uveitis patients revealed significantly elevated levels of growth/differentiation factor–15 (GDF–15) by >48-fold compared to controls (*n* = 16). The whisker plots represent the median and the interquartile range in the distribution. (**B**) Demographic details of the study population. *p*–values were based on the Mann–Whitney U test. Abbreviations: GDF15, growth/differentiation factor-15; CTL, control; CMV–HAU, CMV positive hypertensive anterior uveitis.

As described above, having found significant changes in the inflammation related proteins in the AH of CMV-HAU patients, in these samples, we also examined changes in the levels of GDF-15, a well-recognized inflammatory and stress response marker related to the TGF-β superfamily of proteins by ELISA [17,18,19]. Figure 3A shows a significant (*p* < 0.001; *n* = 16) and robust increase in GDF-15 protein levels by 48-fold compared to the control (non-glaucoma) samples. These AH samples were derived from a different cohort from the same hospital, and the demography details are described in Figure 3B.

**Figure 4:**
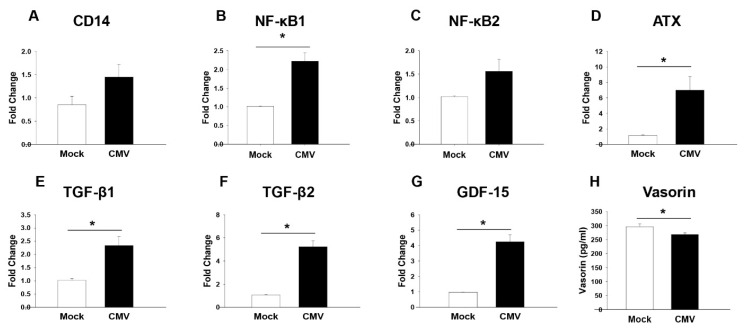
Increased inflammatory markers after CMV infection in human TM cells. TM cells, the key cells involved in the regulation of IOP, were used to understand the increased inflammation-related pathways shown in the aqueous humor proteomic analyses in the CMV hypertensive anterior uveitis. qPCR results (**A**–**G**) and ELISA results (**H**). Inflammation—related genes (NF-κB1, ATX, TGF-β1 and TGF-β2, and GDF-15) were significantly elevated in the qPCR analyses in CMV-infected TM cells. However, the protein expression level of vasorin using ELISA was significantly decreased in the CMV-infected TM cells compared to the mock-infected TM. All experiments were repeated in at least triplicate (*n* = 3, Student’s *t*-test for statistical analysis). Abbreviations: CMV, cytomegalovirus; CD14, cluster of differentiation 14; NF-κB, nuclear factor kappa-light-chain-enhancer of activated B cells; ATX, autotaxin; GDF-15, growth/differentiation factor-15; TGF-β1, transforming growth factor-β1. * *p* < 0.05.

To gain further insight into inflammation in CMV-HAU patients, we determined the inflammatory markers in the CMV-infected human TM cells by qRT-PCR analysis, which are the key cells involved in the regulation of AH outflow through the conventional pathway and IOP [20,21]. As shown in Figure 4, the expressions of NF-kB1, autotaxin (ATX), TGF-β1, TGF-β2, and GDF-15 were significantly elevated in the CMV-infected human TM cells (*p* < 0.05), whereas the levels of vasorin, a well-characterized anti-TGF-β [22,23], in the conditioned media of CMV infected TM cells were significantly decreased compared to the control samples (Figure 4H).

**Figure 5:**
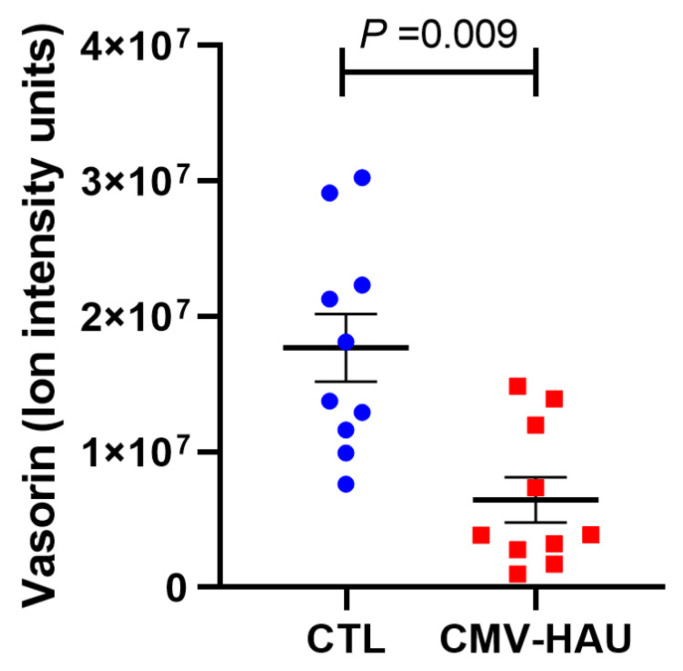
Decreased vasorin levels in the AH of CMV hypertensive anterior uveitis by mass spectrometry. Vasorin levels were significantly decreased in the AH of CMV hypertensive anterior uveitis patients compared to controls by 0.45-fold. Abbreviations: CTL, control; CMV-HAU, CMV hypertensive anterior uveitis.

Interestingly, vasorin was also one of the proteins that was found to be significantly decreased in the AH of CMV-HAU patients compared to the controls based on the LC-MS/MS analysis described above (Table 2). We plotted the individual values of vasorin levels detected in the mass spectrometry analysis, and as shown in Figure 5, there were significant (*p* = 0.009; *n* = 10) decreases in the levels of vasorin in the AH of CMV-HAU patients compared to the control samples.

## 4. Discussion

The main objective of this study was to identify the proteins that exhibit an altered profile in the AH of CMV-positive hypertensive anterior uveitis compared to non-glaucoma controls to understand their possible involvement in the pathogenesis of uveitis glaucoma. This study revealed and identified several proteins (~50 proteins) whose levels were significantly upregulated in the CMV-HAU patients compared to the controls. Importantly, functional analyses based on GO enrichment revealed that the altered AH proteins (based on mass spectrometry analysis) were involved in a variety of biological functions, including complement activation, humoral immune response, proteolysis, phospholipid metabolism, and platelet degranulation, indicating dysregulation and participation of the complement regulated inflammation and activation of the immune response in CMV-HAU-associated ocular hypertension and glaucoma.

Innate immunity such as natural killer cells, monocyte-macrophages, neutrophils, as well as complement system are all suppressed by the ocular immune privilege [24,25]. The eye exhibits little to no expression of the major histocompatibility complex (MHC), which presents viral epitopes for the CD8+ cytotoxic cell. Therefore, the absence of the MHC class I molecule creates a potential immunologic blind spot for viral infection [26], which may explain the frequent recurrence of CMV or HSV-1 infection in immunocompetent patients.

In this study, we found that the complement activation was the most abundantly enriched pathway in the AH of CMV-HAU. Activation of the complement cascade generates mediators that directly damage cells by a consequent formation of the membrane attack complex which attracts and triggers complement receptors culminating into the activation of innate immune cells [27]. Complement system dysregulation was found in patients with glaucoma, as well as those with uveitis [28]. Altered complement activation cascade was shown in the AH proteome of patient with POAG [29]. Elevated levels of C1q, C3, and C5b-9 were also noted in the retina of patients with glaucoma [30]. Especially, increased levels of C3 and factor B, C4, and C5 as well as autoantibodies against ocular proteins were found in AH of idiopathic uveitis [31,32]. The dysregulation of the complement system is thought to play a role in the pathogenesis of autoimmune anterior uveitis [28]. The role of complement activation in the AH outflow facility needs to be investigated in future studies. Interestingly, although, the proteomic analysis of AH of CMV-HAU patients did not show significant changes in the levels of GDF-15, ELISA-based evaluation detected its robust elevation in the AH of CMV-HAU samples relative to the control samples. GDF15 is a peptide hormone, its levels are known to be elevated under tissue injury, inflammation, and pathological conditions, including glaucoma [17,19,33]. This observation together with the elevated levels of complement factors in the AH of CMV-HAU described above indicate an increased inflammatory response in CMV-HAU.

In addition, we showed that CD14, which acts as a co-receptor along with the Toll-like receptor (TLR) 4 in macrophages as part of the innate system helping to detect bacteria in the body by binding lipopolysaccharide (LPS), a pathogen-associated molecular pattern (PAMP) [34], was found to be one of the most elevated proteins (by ~50 fold) in the AH of CMV-HAU compared to the controls (Table 1). This finding suggests that the innate immune system was set into motion in the AH of CMV-HAU.

Antigens entering the anterior chamber induced an anterior chamber-associated immune deviation (ACAID), as part of the immune privilege system in the eye [35]. By ACAID, T-cell mediated immune reaction, the delayed-type hypersensitivity, was actively suppressed, whereas humoral immunity was relatively preserved [26]. In association with this, we found that the humoral immune response mediated by the circulating immunoglobulin was the second enriched biologic pathway in the AH of CMV-HAU (Figure 1).

The ocular microenvironment contains immunosuppressive mediators such as transforming growth factor (TGF)-β, macrophage immigration inhibitory factor, and α-MSH. These cytokines are recognized to influence the microenvironment of various tissues of the ocular anterior chamber and impact their immune response and other activities, including IOP. Among this, TGF-β is one of the cardinal cytokines affecting ocular fibrosis and increasing resistance to the outflow facility [36,37,38,39]. CMV infection induces the secretion of TGF-β, modifying the infected cells and systemic immune reactions to the advantage of virus replication by both upregulating CMV replication and downregulating host immune responses [40]. Interestingly, in the AH of CMV-HAU, GDF-15, which belongs to the TGF-β superfamily proteins, it showed a 40-fold increase compared to the non-glaucoma controls. Additionally, the levels of vasorin, the anti-TGF-β glycoprotein [22,23], were significantly decreased in the AH of CMV-HAU. These finds suggest that CMV-HAU is characterized by the elevation of TGF-β activity, and this could be contributing partly to the clinical characteristics of CMV-HAU, which is known to have mild inflammation in the anterior chamber but to exhibit a robust elevation in IOP.

Finally, in this study, we also recorded increased levels of myocilin by 9-fold in the AH of CMV-HAU compared to the controls (Table 1). Myocilin was highly expressed in the trabecular meshwork, and was detected in AH and stimulated by dexamethasone [41], retinoic acid [42], and TGF-β [41,43]. Although dexamethasone causes overexpression of myocilin, it is not clear that the overexpression by itself is directly associated with IOP elevation [44,45]. The patients in our study were treated by steroid and anti-glaucomatous eyedrops during the follow-up period, and this could have contributed partly to the increased myocilin levels found in the CMV-HAU patients.

### Limitations

There are several limitations to be acknowledged for this study. Since the collection of AH from the CMV-HAU patients in the clinic is a highly invasive procedure, the number of samples analyzed were relatively limited. Second, we used cataract patients as non-glaucoma control subjects undergoing elective surgery. Although considered normal, it may affect the results. In addition, due to the small volumes of AH obtained, sufficient samples were not available for further validation of the mass spectrometry-derived findings by methods such as Western blotting.

## 5. Conclusions

In conclusion, this study demonstrates the changes in the AH proteome composition in human patients with CMV-positive hypertensive anterior uveitis, and the recoded changes in the protein profile reveal complement activation and humoral immunity. Further studies are necessary to validate these findings to understand their precise pathogenic role in the CMV-HAU.

## Figures and Tables

**Table 1:** Significantly upregulated proteins in the aqueous humor of the patients with CMV hypertensive anterior uveitis.

SwissProt Accession	Protein Name	Gene Name	Confidence Score	Mass	Fold Change	SEM	*p*-Value
Q99574	Neuroserpin	SERPINI1	36.52	46,397	107.418	35.202	0.034
P08571	Monocyte differentiation antigen CD14	CD14	221.71	40,678	49.448	19.642	0.014
P02747	Complement C1q subcomponent subunit C	C1QC	96.28	25,985	31.305	7.597	0.001
P06310	Immunoglobulin kappa variable 2–30	IGKV2-30	171.55	13,291	18.993	11.785	0.047
P02671	Fibrinogen alpha chain	FGA	882.32	95,656	15.914	3.252	0.049
P00746	Complement factor D	CFD	371.47	27,529	12.955	2.630	0.021
P08697	Alpha-2-antiplasmin	SERPINF2	780.89	54,873	10.648	3.051	0.029
P98160	Basement membrane-specific heparan sulfate proteoglycan core protein	HSPG2	463.75	479,253	9.577	1.577	0.003
Q99972	Myocilin	MYOC	545.94	57,450	9.492	1.951	0.002
P13671	Complement component C6	C6	426.5	108,367	8.149	1.789	0.027
P07358	Complement component C8 beta chain	C8B	181.35	68,714	8.097	2.285	0.048
P0C0L5	Complement C4-B	C4B	836.96	194,170	8.028	2.454	0.044
P05546	Heparin cofactor 2	SERPIND1	734.9	57,205	7.671	1.750	0.044
P04004	Vitronectin	VTN	690.61	55,069	7.582	1.678	0.044
Q9BSG5	Retbindin	RTBDN	40.8	25,397	7.302	2.058	0.050
P01042	Kininogen-1	KNG1	764.45	72,996	7.016	1.639	0.035
P06396	Gelsolin	GSN	2330.63	86,043	6.891	2.099	0.022
P01031	Complement C5	C5	312.35	189,897	6.741	1.119	0.012
Q9Y6R7	IgGFc-binding protein	FCGBP	399.89	596,443	6.624	2.003	0.034
P02746	Complement C1q subcomponent subunit B	C1QB	135.42	26,933	6.401	1.021	0.007
P02748	Complement component C9	C9	789.9	64,615	6.118	1.195	0.034
P43251	Biotinidase	BTD	223.98	62,006	5.655	2.312	0.046
P19827	Inter-alpha-trypsin inhibitor heavy chain H1	ITIH1	743.03	101,782	5.215	0.933	0.011
P01009	Alpha-1-antitrypsin	SERPINA1	3504.62	46,878	5.167	0.940	0.006
P01700	Immunoglobulin lambda variable 1–47	IGLV1-47	181.44	12,447	4.759	0.767	0.001
P10643	Complement component C7	C7	548.4	96,650	4.472	0.752	0.036
P43652	Afamin	AFM	724.79	70,963	4.327	0.651	0.013
P02647	Apolipoprotein A-I	APOA1	2280.23	30,759	4.140	0.719	0.050
P01011	Alpha-1-antichymotrypsin	SERPINA3	2881.01	47,792	4.126	0.676	0.016
P02649	Apolipoprotein E	APOE	1047.38	36,246	3.775	0.562	0.041
P02768	Albumin	ALB	13,704.76	71,317	3.640	0.640	0.030
Q13449	Limbic system-associated membrane protein	LSAMP	40.52	37,883	3.584	0.559	0.006
P02753	Retinol-binding protein 4	RBP4	516.31	23,337	3.501	0.651	0.048
P05543	Thyroxine-binding globulin	SERPINA7	537.84	46,637	3.490	0.435	0.009
P00751	Complement factor B	CFB	1454.73	86,847	3.411	0.631	0.047
P04196	Histidine-rich glycoprotein	HRG	1350.42	60,510	3.381	0.500	0.000
P01008	Antithrombin-III	SERPINC1	2746.51	53,025	3.057	0.424	0.011
P04406	Glyceraldehyde-3-phosphate dehydrogenase	GAPDH	40.47	36,201	2.760	0.831	0.045
A0A075B6Q5	Immunoglobulin heavy variable 3–64	IGHV3-64	34.36	13,053	2.714	0.366	0.018
P05156	Complement factor I	CFI	548.08	68,102	2.708	0.343	0.000
P48740	Complement C1s subcomponent	MASP1	845.36	78,174	2.529	0.300	0.016
P15924	Desmoplakin	DSP	282.15	334,021	2.518	0.915	0.038
P12259	Coagulation factor V	F5	249.16	252,686	2.377	0.721	0.037
Q6UWP8	Suprabasin	SBSN	145.13	60,562	2.275	0.843	0.003
P60174	Triosephosphate isomerase	TPI1	60.32	31,057	2.241	0.813	0.037
A0A0B4J1V0	Immunoglobulin heavy variable 3–15	IGHV3-15	345.55	13,089	2.209	0.251	0.037
P10909	Clusterin	CLU	1756.16	53,031	2.203	0.372	0.040
P01019	Angiotensinogen	AGT	955.58	53,406	2.115	0.473	0.035
P14923	Junction plakoglobin	JUP	138.53	82,434	2.110	0.783	0.016
P00450	Ceruloplasmin	CP	4829.27	122,983	2.076	0.208	0.008

Footnote: The sample number for the CMV uveitis and non-glaucoma control patients was 10 per group.

**Table 2:** Significantly downregulated proteins in the aqueous humor of the patients with CMV hypertensive anterior uveitis.

SwissProt Accession	Protein Name	Gene Name	Confidence Score	Mass	Fold Change	SEM	*p* Value
P51884	Lumican	LUM	172.21	38,747	0.262	0.031	0.002
P98164	Low-density lipoprotein receptor-related protein 2	LRP2	90.94	540,376	0.343	0.042	0.001
Q92823	Neuronal cell adhesion molecule	NRCAM	143.1	144,655	0.356	0.058	0.001
O00533	Neural cell adhesion molecule L1-like protein	CHL1	43.02	136,070	0.370	0.053	0.000
P07451	Carbonic anhydrase 3	CA3	41.84	29,824	0.375	0.069	0.003
P78509	Reelin	RELN	27.64	394,980	0.392	0.079	0.023
Q16610	Extracellular matrix protein 1	ECM1	123.22	62,232	0.393	0.055	0.002
O60938	Keratocan	KERA	63.67	40,882	0.402	0.185	0.030
Q99435	Protein kinase C-binding protein NELL2	NELL2	38.93	96,359	0.405	0.113	0.016
Q12841	Follistatin-related protein 1	FSTL1	34.61	36,103	0.446	0.081	0.012
Q6EMK4	Vasorin	VASN	211.36	72,751	0.448	0.063	0.009
Q5D862	Filaggrin-2	FLG2	193.17	249,296	0.482	0.050	0.019
Q14515	SPARC-like protein 1	SPARCL1	363.95	76,017	0.486	0.053	0.004
P24592	Insulin-like growth factor-binding protein 6	IGFBP6	55.43	26,219	0.492	0.116	0.027
Q9BY67	Cell adhesion molecule 1	CADM1	90.24	48,935	0.492	0.066	0.009

Footnote: The sample number for the CMV uveitis and non-glaucoma control patients was 10 per group.

## Data Availability

Authors will make all data available upon request.

## Supplementary Materials

The following supporting information can be downloaded at: https://www.mdpi.com/article/10.3390/jcm11092337/s1, Table S1. Sequence for forward and reverse primer sets used in real-time qPCR. Table S2. Clinical characteristics of the CMV anterior uveitis patients involved in the study. Table S3. The functional enrichment analysis and the predicted genes related to the corresponding function.
