# Supplementary material for: Increased Complement-Associated Inflammation in Cytomegalovirus-Positive Hypertensive Anterior Uveitis Patients Based on the Aqueous Humor Proteomics Analysis
Source: J Clin Med. 2022 Apr 22;11(9):2337. doi: 10.3390/jcm11092337 (PMC9101446; doi:10.3390/jcm11092337)
Supplement: Supplementary file 1 [file jcm-11-02337-s001.zip › jcm-1674328-supplementary.pdf]

Increased Complement-associated Inflammation in Cytomegalovirus-Positive Hypertensive Anterior Uveitis Patients based on the Aqueous Humor Proteomics Analysis

Jin A Choi, MD. Ph.D.[1,#], Hyun-hee Ju[1], Jiyoung Lee[2],MD., Ju-Eun Kim[3],

Soon-Young Paik.PhD.[3], Nikolai P. Skiba,Ph.D.,[4] Ponugoti Vasantha Rao, Ph.D.[4]

1. Department of Ophthalmology, College of Medicine, St. Vincent's Hospital, The Catholic University of Korea, Seoul Korea.
2. Department of Ophthalmology, College of Medicine, Daejon St. Mary's Hospital, The Catholic University of Korea, Seoul Korea.
3. Department of Microbiology, College of Medicine, The Catholic University of Korea, Seoul, Republic of Korea.
4. Department of Ophthalmology, Duke University School of Medicine, Durham, NC. USA.

Article type: Original

# Correspondence

Jin A Choi, MD. Ph.D.

Email: jinah616@catholic.ac.kr

Table S1. Sequence for forward and reverse primer sets used in real-time qPCR.

Gene Name	Forward Primer	Reverse Primer	Product Size
CD14	CTCAACCTAGAGCCGTTTCT	AGCTGAGCAGGAACCTGTG	125bp
NF-kB1	GTGGTGCCTCACTGCTAACT	GGGAAATTGTCAGCAGGCTA	94bp
NF-kB2	TAGCCACAGAGATGGAGGAG	GTGTGTTTCCCGCAAAGGT	93bp
Fibronectin	CTGGCCGAAAATACATTGTAA	CCACAGTCGGGTCAGGAG	113bp
ATX	ACAACGAGGAGAGCTGCAAT	AGAAGTCCAGGCTGGTGAGA	113bp
GDF-15	GTTAGCCAAAGACTGCCACTG	CCTTGAGCCCATTCCACA	105bp
TGF-β1	GAGCCTGAGGCCGACTACTA	GGGTTCAGGTACCGCTTCTC	149bp

Abbreviations: CD14, cluster of differentiation 14; NF-κB, nuclear factor kappa-light-chain-enhancer of activated B cells; ATX, autotaxin; GDF-15, growth/differentiation factor-15; TGF-β1 Transforming growth factor-β1.

Table S2. Clinical characteristics of the CMV anterior uveitis patients involved in the study

	**Controls (n=10)**	**CMV hypertensive anterior uveitis (n=10)**	***P* value**
Gender (Male: Female)	4:6	8:2	0.170
Average age, year	65.8 ± 6.9<br>(range: 57-81)	53.1 ± 15.3<br>(range: 35-80)	0.023
Peak preoperative IOP, mmHg	11.7 ± 1.9<br>(range: 9-13)	31.5 ± 9.4<br>(range: 21-52)	< 0.001
Number of anti-glaucoma medication, number	N/A	2.1 ± 1.0	
Corneal endothelial cell count, cell/mm2	2690.9 ± 450.9	1905.3 ± 810.1	0.015
MD, dB	N/A	-8.43 ± 6.51<br>(range: -1.3 - -20.5)	**value**
Average RNFLT, um	N/A	78.2 ± 16.2<br>(range: 57-102)	

Abbreviations: IOP, intraocular pressure; MD, mean deviation; RNFLT, retinal nerve fiber layer thickness.

Table S3. The functional enrichment analysis and the predicted genes related to the corresponding function

High level GO category	Genes
Response to stress	C6 DSP SERPIND1 C5 VTN GAPDH C7 C9 KNG1 HRG SERPINC1 APOA1 CLU MASP1 APOE HSPG2 GSN C1QC ALB CD14 FGA SERPINA3 F5 CFI C4B_2 CFB CFD SERPINF2
Response to external stimulus	C6 SERPIND1 C5 VTN GAPDH C7 C9 KNG1 HRG SERPINC1 APOA1 CLU MASP1 APOE GSN C1QC ALB CD14 FGA JUP CFI C4B_2 CFB CFD SERPINF2
Immune system process	C6 DSP C5 VTN GAPDH C7 C9 HRG SERPINC1 APOA1 CLU MASP1 APOE RBP4 GSN C1QC CD14 FGA JUP SERPINA3 CFI C4B_2 CFB CFD
Immune response	C6 DSP C5 VTN GAPDH C7 C9 HRG SERPINC1 APOA1 CLU MASP1 APOE GSN C1QC CD14 FGA JUP SERPINA3 CFI C4B_2 CFB CFD
Regulation of response to stimulus	MYOC C6 C5 VTN C7 C9 KNG1 HRG SERPINC1 APOA1 CLU MASP1 APOE C1QC CD14 FGA JUP CFI C4B_2 CFB CFD SERPINF2
Regulation of biological quality	MYOC AFM DSP SERPIND1 VTN GAPDH C7 KNG1 HRG SERPINC1 APOA1 CLU SERPINA7 APOE RBP4 GSN ALB FGA JUP SERPINA3 F5 SERPINF2
Immune effector process	C6 DSP C5 VTN C7 C9 APOA1 CLU MASP1 RBP4 GSN C1QC CD14 JUP SERPINA3 CFI C4B_2 CFB CFD
Regulation of immune system process	C6 C5 VTN C7 C9 HRG APOA1 CLU MASP1 APOE RBP4 C1QC CD14 FGA CFI C4B_2 CFB CFD
Regulation of molecular function	ITIH1 SERPIND1 C5 VTN GAPDH KNG1 HRG SERPINC1 APOA1 CLU SERPINA7 APOE GSN SERPINI1 JUP SERPINA3 C4B_2 SERPINF2
Response to biotic stimulus	C6 C5 GAPDH C7 C9 HRG CLU MASP1 APOE GSN C1QC CD14 FGA CFI C4B_2 CFB CFD
Biological process involved in interspecies interaction between organisms	C6 C5 GAPDH C7 C9 HRG CLU MASP1 APOE GSN C1QC CD14 FGA CFI C4B_2 CFB CFD
Regulation of multicellular organismal process	DSP C5 VTN GAPDH KNG1 HRG SERPINC1 APOA1 CLU APOE RBP4 HSPG2 C1QC CD14 FGA JUP SERPINF2
Response to other organism	C6 C5 GAPDH C7 C9 HRG CLU MASP1 APOE GSN C1QC CD14 FGA CFI C4B_2 CFB CFD
Cellular localization	DSP KNG1 HRG APOA1 CLU APOE GSN ALB CD14 FGA JUP SERPINA3 F5 CFD SERPINF2
Regulation of localization	MYOC DSP C5 VTN HRG APOA1 CLU APOE RBP4 GSN CD14 FGA JUP C4B_2

Anatomical structure morphogenesis	MYOC DSP C5 VTN HRG APOA1 CLU APOE RBP4 HSPG2 FGA JUP SERPINF2
Biological adhesion	MYOC DSP VTN KNG1 HRG APOA1 GSN SERPINI1 FGA JUP LSAMP SERPINF2
Activation of immune response	C6 C5 VTN C7 C9 CLU MASP1 C1QC CFI C4B_2 CFB CFD
System process	MYOC DSP KNG1 APOA1 APOE RBP4 GSN FGA JUP SERPINA3 F5 SERPINF2
Cell adhesion	MYOC DSP VTN KNG1 HRG APOA1 GSN SERPINI1 FGA JUP LSAMP SERPINF2
Regulation of signaling	MYOC VTN HRG APOA1 CLU APOE RBP4 CD14 FGA JUP SERPINF2
Cellular component biogenesis	MYOC C9 HRG APOA1 CLU APOE GSN FGA JUP F5 SERPINF2
Regulation of developmental process	MYOC C5 HRG APOA1 APOE RBP4 HSPG2 C1QC FGA JUP SERPINF2
